# Short-term outcomes for intracorporeal vs. extracorporeal anastomosis in laparoscopic right hemicolectomy for colonic cancer—a prospective cohort study (ICEA-study)

**DOI:** 10.1007/s00384-025-04882-1

**Published:** 2025-04-08

**Authors:** Emilie Schultz Hougaard, Benedicte Schelde-Olesen, Issam Al-Najami, Thomas Buchbjerg, Benjamin Schnack Brandt Rasmussen, Lasse Bugge, Thomas Kolbro, Sören Möller, Mark Bremholm Ellebæk

**Affiliations:** 1 Department of Surgery, Odense University Hospital, Odense, Denmark; 2 OPEN, Odense University Hospital, Odense, Denmark; 3 Department of Clinical Research, University of Southern Denmark, Odense, Denmark; 4 Department of Radiology, Odense University Hospital, Odense, Denmark; 5 Centre for Clinical Artificial Intelligence (CAI-X), Odense, Denmark

**Keywords:** Colonic cancer, Intracorporeal anastomosis, Extracorporeal anastomosis

## Abstract

**Purpose:**

The purpose of this study is to compare short-term outcomes and 1-year incisional hernia rates between intracorporeal anastomosis (IA) and extracorporeal anastomosis (EA) in laparoscopic right hemicolectomy for management of right-sided colonic cancer. The primary outcome was the complication rate assessed by the comprehensive complication index (CCI). Secondary outcomes included time to bowel movement, length of hospital stay, 30-day readmission rate, early warning scores, and 1-year incisional hernia rate.

**Method:**

This was a single-center, prospective cohort study. Patients with right-sided colonic cancer eligible for laparoscopic surgery with primary anastomosis were consecutively included. Patients included in the first period underwent EA, while those in the second underwent IA. Clinical data were collected during the hospital admission up to 30 days postoperatively. Complications were evaluated by the CCI. A routine 1-year CT-scan was used to assess hernias.

**Results:**

One hundred three patients (51 in the EA and 52 in the IA groups) were included. Demographics were similar between the two groups. No significant difference in the CCI-score was found (EA: 17.9 (23.9) vs. IA: 15.0 (17.4), *p* = 0.85). The mean length of hospital stay was significantly shorter in the IA group (EA 6.6 days, IA 3.9 days, *p* = 0.02). The groups had no significant differences regarding other outcomes, including hernia rates (*p* = 0.12).

**Conclusion:**

Laparoscopic right hemicolectomy with IA significantly reduced the length of hospital stay without increasing complication rates compared to EA.

**Trial registration:**

The study is registered at ClinicalTrials.gov (NCT05039762).

**Supplementary Information:**

The online version contains supplementary material available at 10.1007/s00384-025-04882-1.

## Introduction

Laparoscopic right hemicolectomy remains the standard treatment for right-sided colon cancer. This minimally invasive procedure has demonstrated significant benefits, including reduced postoperative pain, shorter hospital stays, and a lower incidence of surgical site infections, all of which contribute to an improved quality of life for patients compared to traditional open surgery [1]. Over time, surgical techniques have evolved, focusing on minimizing invasiveness and reducing surgical stress. In this context, laparoscopic right hemicolectomy with intracorporeal anastomosis (IA) has emerged as a promising approach.

Previous studies have shown that IA offers advantages over extracorporeal anastomosis (EA) regarding shorter hospital stays and shorter time to bowel recovery without an increase in the risk of severe complications or negatively impacting the oncological outcomes [2–8]. A review from 2019 supports these findings, noting a significant reduction in the overall complication rate in the IA group compared to the EA group. Additionally, lower incidences of anastomotic leakage, surgical site infection, and incisional hernias were reported following hemicolectomy with IA when compared to EA [9].

A randomized clinical trial evaluating IA vs EA in laparoscopic right colectomies utilized the comprehensive complication index (CCI) to assess the accumulated severity of complications [10]. This study found a significant 5.9-point difference in favor of IA, alongside quicker bowel recovery and reduced postoperative pain, as indicated by lower visual analogue scale (VAS) scores.

Given that IA is generally considered less invasive than EA, it is hypothesized that the postoperative surgical stress response would be reduced when performing IA compared to EA. Supporting this hypothesis, a randomized trial from 2018 investigating IA in laparoscopic right hemicolectomy [11] found that the surgical stress response measured by CRP and interleukin- 6 was significantly lower in the IA group.

Despite the promising outcomes associated with IA, its use remains limited according to Danish Colorectal Cancer Group (DCCG) data, where only 5% of the 5941 right hemicolectomies performed between 2014 and 2018 in Denmark used the IA technique [12]. This highlights the need for further research to understand better IA’s safety profile and potential advantages over EA. While some prospective studies have been conducted, a dedicated trial comparing these techniques is still needed to consolidate the available knowledge.

This study aims to compare the outcomes of laparoscopic right hemicolectomies with IA versus EA in patients diagnosed with colon cancer. Specifically, we sought to evaluate differences in overall complication rates (CCI), time to bowel recovery, length of hospital stay, postoperative pain, surgical stress response, and the incisional hernia rate.

## Method

### Ethics approval and trial registration

The ICEA study received ethical approval from the regional Ethics Committee (S- 20200076) and the Danish Data Protection Agency (20/35765). It was registered on ClinicalTrials.gov under the identifier NCT05039762.

### Study design

This prospective cohort study was conducted as a single-center trial at the Department of Surgery, Odense University Hospital in Svendborg, a tertiary colorectal center. Patients were consecutively included in the period from September 1, 2020, to April 1, 2022, with an initial follow-up of one year. The inclusion period was divided into two. Patients recruited during the first period of the study (September 2020 to August 2021) received EA, while those enrolled during the second period (September 2021 to March 2022) were treated with intracorporeal anastomosis (IA).

The study adhered to the reporting recommendation outlined by the Equator Network (https://www.equator-network.org/) following the STROBE (Strengthening the Reporting of Observational Studies in Epidemiology) guidelines [13].

### Study population and inclusion

Patients diagnosed with right-sided colonic cancer, scheduled for elective laparoscopic right hemicolectomy with primary anastomosis, evaluated at multidisciplinary team (MDT) conference, were considered eligible for inclusion. Eligible individuals were required to have an Eastern Cooperative Oncology Group (ECOG) [14] performance status of 0–2 and a preoperative CT scan indicating a T1-T3M0 tumor stage [15].

Patients under the age of 18, as well as pregnant or mentally incompetent patients, were not eligible for inclusion. Patients who had undergone acute surgery due to for example due occluding tumor, bowel obstruction or perforations prior to their scheduled elective surgery were excluded from the study.

Certified colorectal surgeons conducted Patient recruitment and inclusions in the outpatient clinic. All potential participants were provided with oral and written information about the study before enrollment. Those who declined participation underwent right hemicolectomy with extracorporeal anastomosis (EA) as per the standard protocol of the department.

### Intervention

All procedures were performed by experienced colorectal surgeons, each certified at the national level and proficient in both anastomosis techniques.

In hemicolectomy with EA, the dissection was performed laparoscopically using the medial to lateral approach as previously described [16]. After adequate mobilization of the right hemi colon and ligation of the associated vessels (including the ileocolic pedicle, right colic pedicle, and the right branch of the middle colic pedicle), the specimen was extracted through an upper midline or horizontal in the upper right quadrant. The small bowel and the transverse colon were then divided using a TLC 75-mm linear stapler, Ethicon (J&J MedTech), and a side-to-side iso-peristaltic ileocolic anastomosis was then hand-sewn.

For laparoscopic hemicolectomy with IA, the vessels’ dissection and ligation were performed similarly. The bowel was transected with a laparoscopic linear automated Signia stapler (Medtronic) 60-mm purple cartridge, the ileum and transverse colon were then joined through an enterotomy in the ileum and transverse colon with the same laparoscopic stapler, and the enterotomy was closed with a running laparoscopic suturing [16]. The specimen was extracted via a Pfannenstiel incision.

After surgery, the patients were admitted to the surgical department for postoperative care. All patients were treated according to an Enhanced Recovery After Surgery (ERAS) protocol [17]. Criteria for discharge included the resumption of oral intake, return of bowel function, pain control by self-administered medication, autonomous urinary function and satisfactory mobility, as assessed by the physiotherapist at the department.

### Outcomes

The study’s primary outcome was the overall complication rate within 30 days, assessed using the CCI.

Secondary outcomes included the following:

- Time to bowel recovery is measured by the first passage of flatus, along with the length of hospital stay in days post-surgery and the 30-day readmission rate.
- Postoperative pain was evaluated using the visual analogue scale (VAS) [18], and the surgical stress response was measured by levels of C-reactive protein (CRP), leucocytes, and the early warning score (EWS) during the four days following surgery.
- Incisional hernia rate corresponding to Pfannenstiel, horizontal, or midline incisions was evaluated by a trained radiologist on CT scans carried out 1 year after the surgery. Port site hernias were not included in this study. Each hernia was measured at its widest point on axial slices (width) and sagittal slices (height), and all measurements were reported in millimeters.

### Data collection and management

Data were prospectively gathered during outpatient consultations before surgery, immediately after the procedure, and during daily rounds conducted by nurses and surgeons. Postoperative complications were documented based on chart entries by healthcare providers, including diagnoses and prescribed medications recorded during the hospital stay.

Complications were evaluated by the comprehensive complication index (CCI) [19, 20] based on the Clavien Dindo classification of postsurgical complications [21, 22]. Throughout the hospital stay, pain levels were assessed using the visual analogue scale (VAS), and various physiological parameters (respiratory rate, oxygen saturation, systolic blood pressure, pulse rate, temperature, and level of consciousness) were monitored three times daily. Blood samples were taken once per day to measure CRP levels and leucocyte counts. The research team gathered all relevant data from forms completed by the surgeons and from the patient’s medical records. The incisional hernia rate was evaluated by a trained radiologist based solely on the CT scan performed 1 year after surgery as a part of the recommended surveillance program for cancer patients [23]. The data were stored in a Research Electronic Data Capture (RedCap ®), hosted by the Open Patient Data Explorative Network (OPEN), at Odense University Hospital [24].

### Power calculation and statistical analysis

To detect a clinically relevant difference in the overall complication rate of 7 points on CCI with the following assumptions 80% power, alpha of 0.05, and expected dropout of 5%, 51 patients were needed in each group.

Dichotomous outcomes are reported as counts and proportions, while numerical outcomes are reported as means with standard deviations. We compared dichotomous outcomes between treatment groups by Fisher’s exact test. Numerical outcomes measured multiple times were compared by linear mixed effect regression, taking repeated measurements from the same patient into account by including a random intercept. *p*-values below 0.05 were considered statistically significant. Analyses were performed using Stata 17.

## Results

**Fig. 1:**
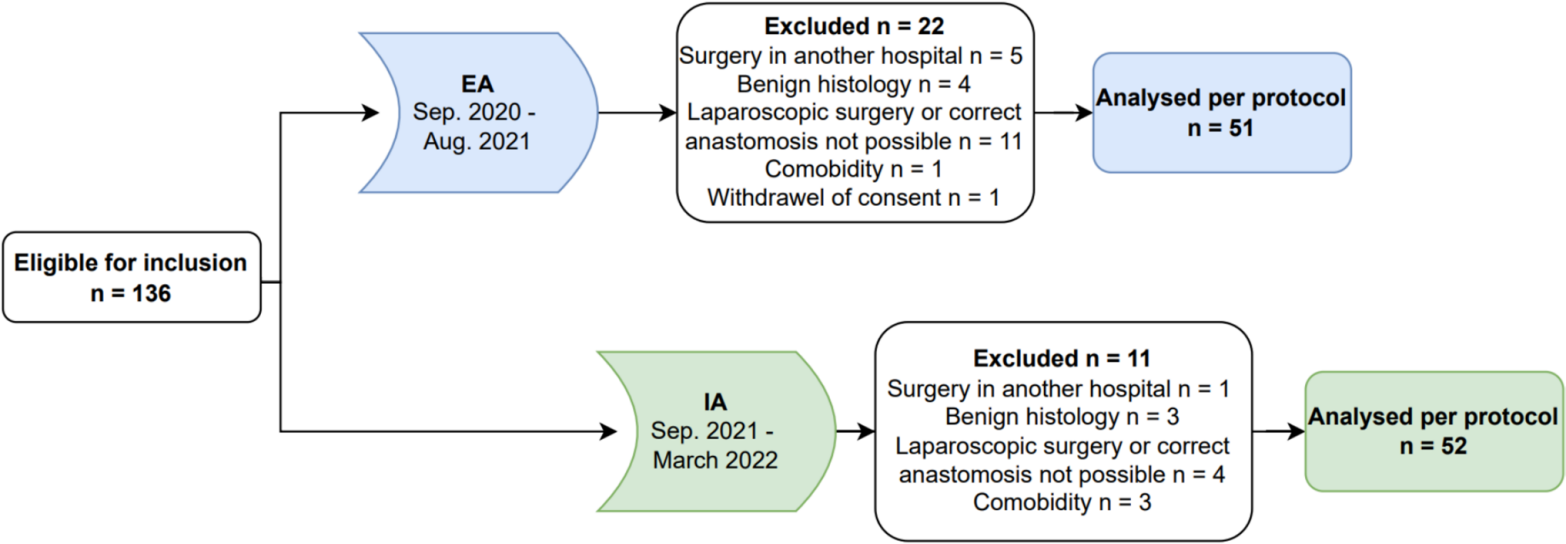
Patient flow. EA: extracorporeal anastomosis, IA: intracorporeal anastomosis

Between September 1, 2020, and April 1, 2022, 136 patients were initially deemed eligible for inclusion. Of these, 33 patients were excluded: 22 from the EA group and 11 from the IA group (Fig. 1). Consequently, 103 patients remained for the final analysis, with 51 in the EA group and 52 in the IA group.

There was a significant difference in the performance status(*p* = 0.04) and number of smokers (*p* = 0.02) in the two groups, but other characteristics were similar (Table 1).

**Table 1:** Demographics and clinical characteristics

	EA, *n* = 51	IA, *n* = 52	*p*-value
Females, *n* (%)	28 (55%)	27 (52%)	0.76
Age, mean (SD)	73.5 (13.7)	76.3 (8.7)	0.37
BMI, mean (SD)	25.8 (5.5)	27.1 (4.1)	0.11
ECOG performance status, *n* (%)			0.04
0	28 (55%)	41 (79%)	
1	17 (33%)	10 (19%)	
2	5 (10%)	1 (2%)	
3	1 (2%)	0 (0%)	
ASA score, *n* (%)			0.52
1	4 (8%)	2 (4%)	
2	28 (55%)	34 (65%)	
3	18 (35%)	16 (31%)	
4	1 (2%)	0 (0%)	
Previous abdominal surgery, *n* (%)	23 (45%)	24 (46%)	0.91
Smoker, *n* (%)			0.02
Yes	6 (12%)	2 (4%)	
No	17 (33%)	31 (60%)	
Previous	28 (55%)	19 (37%)	
Weekly alcohol intake, *n (%)*			
0	15 (29%)	10 (19%)	
< 7 (women)/14 (men) units	30 (59%)	39 (75%)	
> 7 (women)/14 (men) units	6 (12%)	3 (6%)	
Tumor localization, *n* (%)			0.61
Caecum	12 (24%)	16 (31%)	
Ascending colon	24 (47%)	19 (37%)	
Hepatic flexure	6 (12%)	9 (17%)	
Transverse colon	9 (18%)	8 (15%)	

*EA* extracorporeal anastomosis, *IA* intracorporeal anastomosis, *BMI* body mass index, *ECOG* Eastern Cooperative Oncology Group, *ASA* American Society of Anesthesiologists

**Fig. 2:**
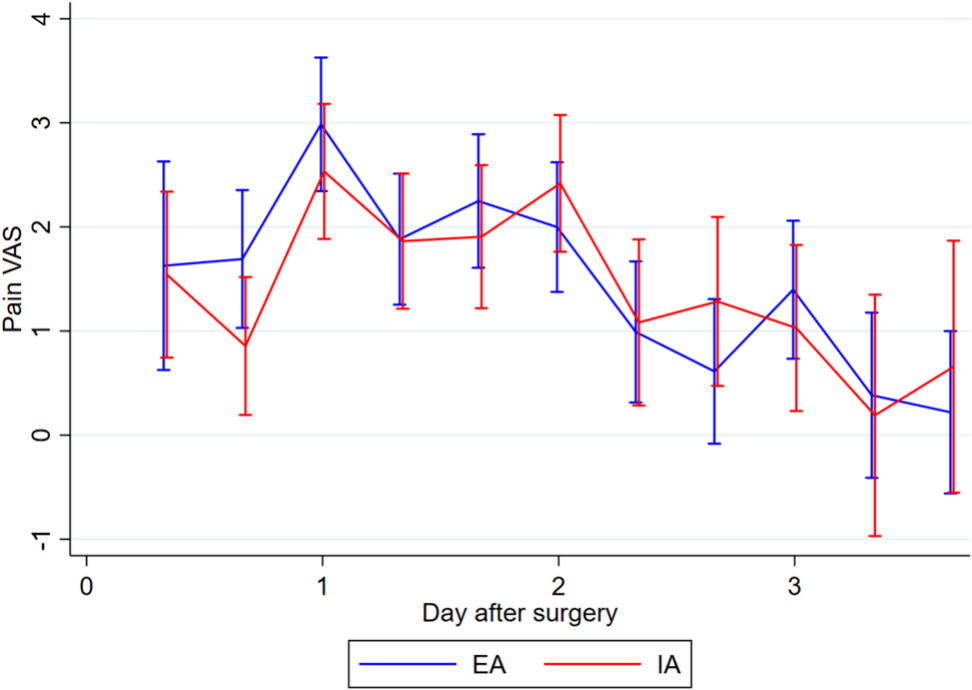
Pain measured on a visual analogue scale (VAS) from 1 to 10 in the first four postoperative days

**Fig. 3:**
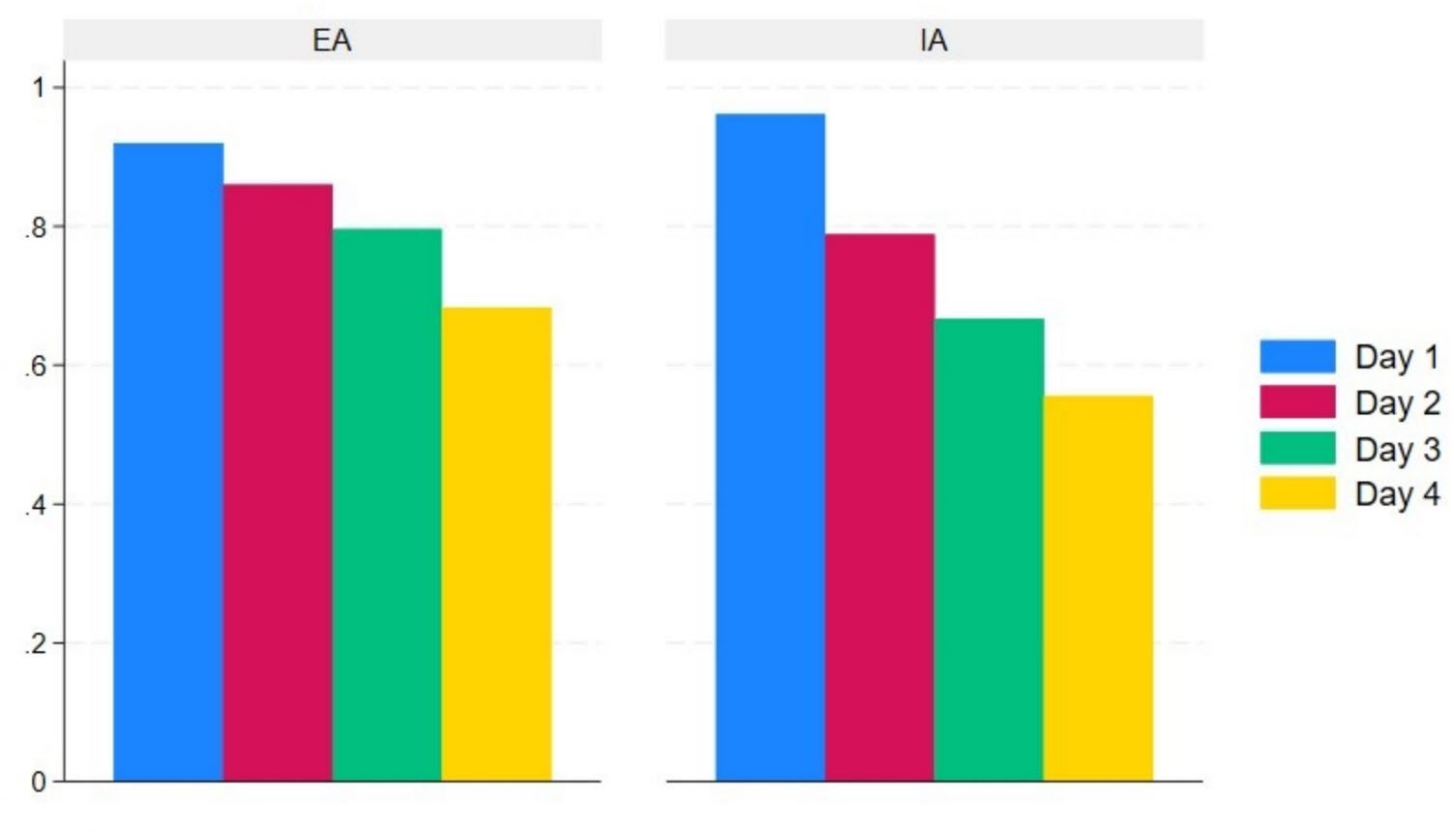
Proportion in percentages of hospital-admitted patients needing opioids on the first four postoperative days

**Fig. 4:**
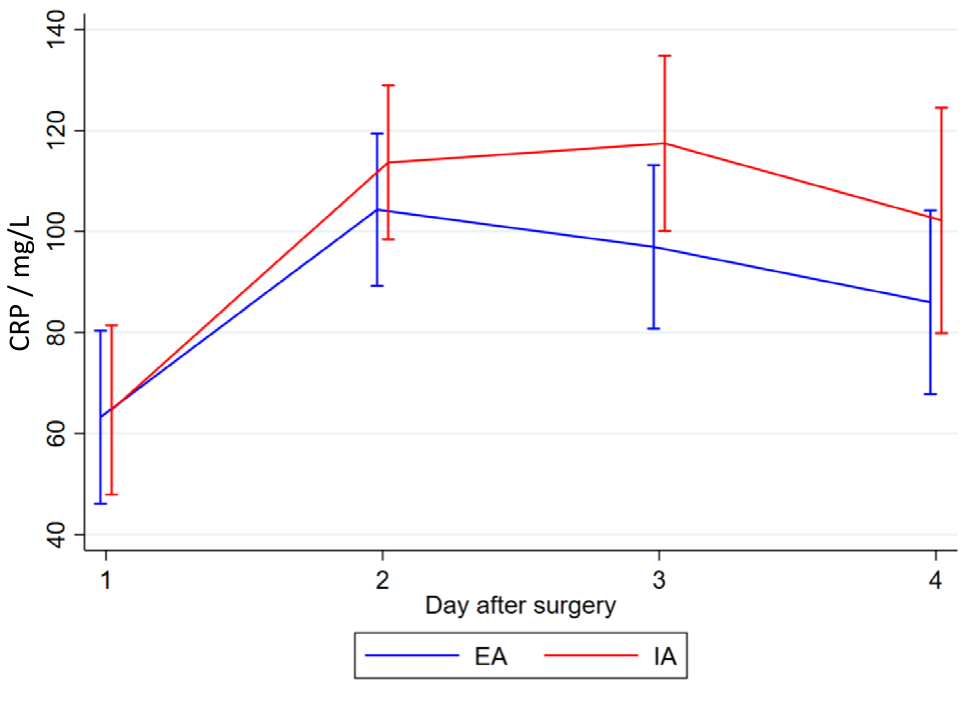
CRP (C-reactive protein) measured in daily blood samples during the first four postoperative days

**Fig. 5:**
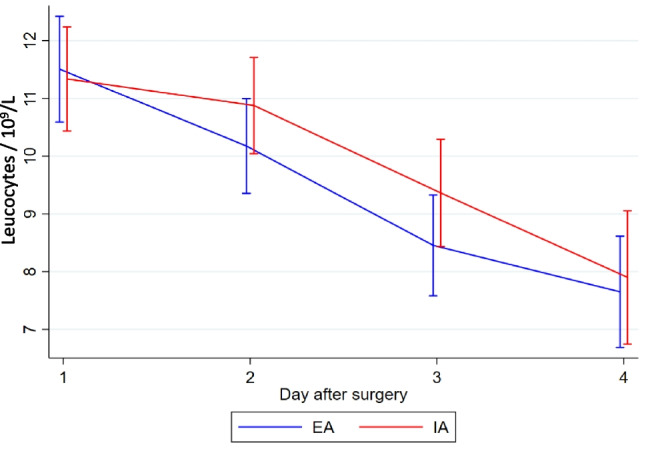
Leucocytes measured in daily blood samples during the first four postoperative days

**Fig. 6:**
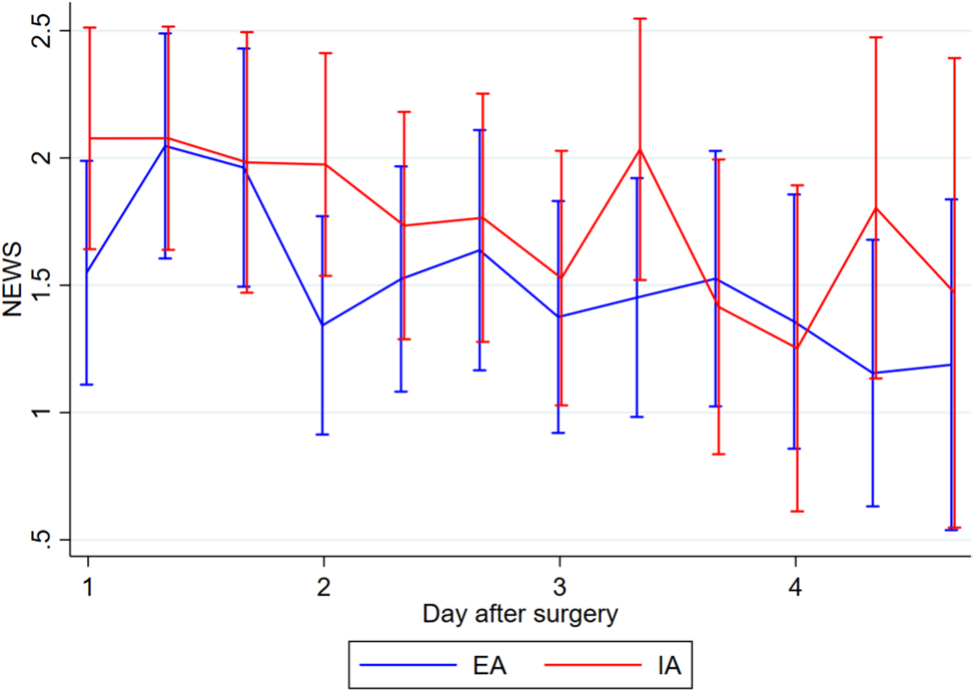
Early warning score (EWS) measured three times a day during the first four postoperative days

There was no statistically significant difference in the overall complication rate between EA and IA (mean (SD) 17.9 (23.9) vs. 15.0 (17.4), *p* = 0.85) (Table 2). One patient from the EA group experienced an anastomotic leak grade C based on the definition suggest by Rahbari et al. [25] requiring a temporary ileostomy, while 1 patient from the IA group experienced an anastomotic leak, which was surgically repaired and preserved. Two patients from the EA group and 1 from the IA group experienced fascia dehiscence graded 3b based on the Clavien Dindo classification of postsurgical complications [21, 22]. Three patients from the EA group developed wound infections, 2 patients with the wound opened bedside, grade 1 complication based on Clavien Dindo [21, 22], and 1 patients with the wound surgically revised graded 3b according to Clavien Dindo [21, 22]. One patient from the EA group developed an intra-abdominal abscess diagnosed based on a CT-scan in relation to the anastomotic site, although it was treated non-surgical with antibiotics. One patient from the IA group developed an intra-abdominal abscess in relation to the port site, which was treated laparoscopically. Time to first flatus was similar in the two groups (mean (SD) EA: 36.8 h (22.5), IA: 37.5 h (22.1), *p* = 0.80). The mean length of hospital stay was significantly shorter in the IA group (EA 6.6 days, IA 3.9 days, *p* = 0.02). The shorter hospital stay in the IA group did not result in a higher readmission rate within 30 days of surgery. In the EA group, 8 patients were readmitted, compared to 7 in the IA group. We found no statistically significant difference in the level of postoperative pain between the groups on the individual days (Fig. 2) or the need for pain relief using opioids (Fig. 3). The same was the case for surgical stress response evaluated by CRP (Fig. 4), leucocytes (Fig. 5), and EWS (Fig. 6). A 1-year follow-up CT scan was carried out on 48 patients in the EA group and 45 in the IA group. Three patients (EA:2, IA: 1) were lost to follow-up. Unfortunately, it was not possible to further specify the reason why these patients did not get a 1-year follow-up CT-scan performed.

**Table 2:** Incisional hernias outcomes

	EA	IA
Incisional hernia rate, *n* (%)	12 (23.5%)	5 (9.6%)
Type of incision		
Horizontal*, n*	10	–
Midline, *n*	2	1
Pfannenstiel, *n*	–	4
Content		
None, *n*	10	2
Intraabdominal fat, *n*	2	2
Soft tissue change, *n*	0	1
Size		
Width in mm, mean (SD)	34 (16)	27 (11)
Height in mm, mean (SD)	20 (16)	22 (13)

All hernias were measured at their widest point, in terms of width on axial slices and height on sagittal slices, reported in millimeters

Two IA patients died before the 1-year scan, and for four patients, the reason for the missing scan was unknown (EA: 1 patient, IA: 3 patients). Incisional hernias were observed in 12 patients (23.5%) from the EA group. Of these, 9 hernias corresponded to a horizontal incision, while 3 corresponded to a midline incision. In the IA group, incisional hernias were observed in 5 patients (9.6%), where 4 corresponded to the Pfannenstiel incision, and 1 corresponded to a midline incision, which was performed instead of a Pfannenstiel in this specific patient in order to reuse a previous midline incision (Table 2). The difference in incisional hernia rates between the EA and IA groups was insignificant (*p* = 0.12) (Table 3). As of January 6, 2025, none of the total 17 patients with incisional hernias evaluated at the 1-year CT scan have had surgery for their incisional hernia.

**Table 3:** Short-term outcomes

	EA	IA	*p*-value
Duration of surgery/minutes, mean (SD)	166 (40)	207 (53)	** < 0.001**
Length of incision (fascia)/cm, mean (SD)	8.9 (0.26)	8.5 (0.21)	0.21
Overall complication rate, CCI score, mean (SD)	17.9 (23.9)	15.0 (17.4)	0.85
Time to bowel recovery/hours, mean (SD)	36.8 (22.5)	37.5 (22.1)	0.80
Length of hospital stay/days, mean (SD)	6.6 (8.6)	3.9 (2.8)	**0.02**
Re-admissions within 30 days, *n* (%)	8 (15.7)	7 (13.5)	0.79

Bold values indicate *p*-values that are statistically significant at the 0.05 level

## Discussion

This prospective study found no difference in the overall complication rate between laparoscopic right hemicolectomy with EA and IA. However, the mean length of hospital stay was significantly shorter in the IA group by one day. No other outcomes demonstrated statistically significant differences between the two anastomotic techniques.

Since the commencement of this study, several other studies have been published, offering additional insights. Overall, these studies suggest that IA is comparable to or superior to EA in many aspects. Several studies have reported similar complication rates between the two anastomotic techniques [26–41], as well as similar overall and recurrence-free survival (3–5 years) [27, 35, 42]. Regarding length of hospital stay, some studies indicate that IA is either equal [27, 32, 34, 36] or superior [28–31, 33, 35, 39–41, 43] to EA.

When interpreting the results from our study, the analysis of the reduced duration of hospitalization in the (IA) group necessitates careful consideration. Given that this was the sole statistically significant parameter, prudence is advised in concluding that a potential risk of a conflict of interest may exist. This conflict could be driven by a preference for the Interventional Analysis technique, consequently lowering the threshold for patient discharge. However, during the study period, the objective discharge criteria at the Department of Surgery of Odense University Hospital in Svendborg remained unchanged. The results regarding readmission do not suggest premature discharge in the IA group, as there was no significant difference in the readmission rate between the two groups.

Regarding bowel function recovery, most recent studies indicate that IA results in a shorter recovery time than EA [26, 29, 31, 32, 34, 38, 41]. Some studies report on postoperative pain, where most find a lower pain level in IA [38, 39, 41, 44]. Finally, the incisional hernia rate is reported to be lower or equal in IA compared to EA [28, 36, 40, 42]

The outcome assessed in this study was the surgical stress response. Given the minimally invasive nature of laparoscopic colon resection with intracorporeal anastomosis (IA), we hypothesized that IA would result in a lower surgical stress response and reduced postoperative pain compared to extracorporeal anastomosis (EA). However, our findings did not support this hypothesis. We found no significant difference in the surgical stress response, and there was a tendency for a higher stress response in the IA group. Furthermore, postoperative pain levels were similar between the two groups. However, these findings could be influenced by a change in surgical protocol during the study period. Complete mesocolic excision (CME) was introduced in our department at the beginning of the IA inclusion period. Most IA patients were, therefore, subjected to a more extensive excision than the patients in the EA group, which could explain, to some extent, the longer surgical time in the IA group. The introduction of CME is a considerable limitation of this study due to the possible increased surgical trauma from CME, it may have masked the hypothesized lower surgical stress response in IA compared to EA. This makes our surgical stress response results challenging to interpret with certainty. Few studies in the literature have directly addressed the surgical stress response of IA and EA. One study reported a significantly higher white blood cell count and higher CRP levels on the first postoperative days in patients who underwent IA, although these differences were not statistically significant compared to the EA group [34].

In another study, it was concluded, based on various inflammatory and metabolic markers, that the stress response following IA was lower than after EA [45]. However, because the IA anastomosis is performed within the abdomen, it is possible that the risk of bacterial contamination could be higher than with EA. A study showed that IA was associated with a higher risk score based on a combination of the dose of bacterial contamination and the virulence of the isolated bacteria in culture from intraperitoneal lavage around the anastomotic site when compared to EA [46]. These findings highlight the importance of considering preventive measures to reduce the risk of infection when performing the IA technique. One study reported a higher incidence of abdominal infections after performing the IA technique when compared to EA, however, the overall complication rate was found to be similar [47]. A recent meta-analysis, which included data from seven randomized controlled trials, supports the conclusion that IA and EA are equal regarding the overall postoperative complication rate in line with our study. However, in contrast to our results, they found no difference in the length of hospital stay between the two groups [48].

The growing adoption of IA over EA brings up important economic considerations. In our healthcare systems, the cost-effectiveness of a new procedure is crucial to the possible implementation. An Italian research group investigated the costs associated with both anastomotic techniques [49] and found overall costs were similar, although IA was associated with higher intraoperative expenses compared to EA.

A key strength of our study is its prospective design within a field where most studies are retrospective. Additionally, all surgeries were performed by certified colorectal surgeons, ensuring high expertise in the procedures.

However, there are some limitations to consider. One is the consecutive inclusion period in two phases, starting with EA patients prior to IA patients, which introduces the risk of potential temporal bias due to possible changes in department routines and patient care. Over time, surgeons may have enhanced their surgical proficiency or advanced their techniques, which could have influenced the outcomes.

However, in order to minimize bias, protocols were closely monitored and guidelines for postoperative care remained consistent throughout the study. The introduction of complete mesocolic excision (CME) during the study is an example of an unforeseen protocol change.

We also acknowledge the limitation of not having blinding in this study. Due to the consecutive patient inclusion and the need to ensure optimal management, it was not feasible to blind the doctors involved in postoperative care.

## Conclusions

This study supports the safety of implementing right hemicolectomy with IA as a treatment for right-sided colonic cancer. Our findings indicate that right hemicolectomy with IA significantly shortens the length of hospital stay without increasing the overall complication risk compared to EA.

## Supplementary Information

Below is the link to the electronic supplementary material.

Supplementary file1 (DOCX 31 KB)

## Data Availability

No datasets were generated or analysed during the current study.
